# Molecular Epidemiology of SARS-CoV-2 in Diverse Environmental Samples Globally

**DOI:** 10.3390/microorganisms9081696

**Published:** 2021-08-10

**Authors:** Ariful Islam, Md. Abu Sayeed, Md. Abul Kalam, Jinnat Ferdous, Md. Kaisar Rahman, Josefina Abedin, Shariful Islam, Shahanaj Shano, Otun Saha, Tahmina Shirin, Mohammad Mahmudul Hassan

**Affiliations:** 1 EcoHealth Alliance, New York, NY 10001-2320, USA; sayeed.dvm@gmail.com (M.A.S.); ferdousjinnat90@gmail.com (J.F.); kaisarrahman@ecohealthalliance.org (M.K.R.); drjosefinaabedin@gmail.com (J.A.); sharifdvm51@gmail.com (S.I.); shahanajshano@gmail.com (S.S.); 2 Centre for Integrative Ecology, School of Life and Environmental Science, Deakin University, Burwood, VIC 3216, Australia; 3 Institute of Epidemiology, Disease Control and Research (IEDCR), Dhaka 1212, Bangladesh; tahmina.shirin14@gmail.com; 4 Helen Keller International, Dhaka 1212, Bangladesh; a.kalam724@gmail.com; 5 Department of Microbiology, University of Dhaka, Dhaka 1000, Bangladesh; otun.saha@gmail.com; 6 Faculty of Veterinary Medicine, Chattogram Veterinary and Animal Sciences University, Chattogram 4225, Bangladesh; miladhasan@yahoo.com

**Keywords:** COVID-19, sewage, phylogenetic analysis, mutation, alpha variant

## Abstract

The severe acute respiratory syndrome coronavirus 2 (SARS-CoV-2) has swamped the global environment greatly in the current pandemic. Wastewater-based epidemiology (WBE) effectively forecasts the surge of COVID-19 cases in humans in a particular region. To understand the genomic characteristics/footprints and diversity of SARS-CoV-2 in the environment, we analyzed 807 SARS-CoV-2 sequences from 20 countries deposited in GISAID till 22 May 2021. The highest number of sequences (*n* = 638) were reported in Austria, followed by the Netherlands, China, and Bangladesh. Wastewater samples were highest (40.0%) to successfully yield the virus genome followed by a 24 h composite wastewater sample (32.6%) and sewage (18.5%). Phylogenetic analysis revealed that SARS-CoV-2 environmental strains are a close congener with the strains mostly circulating in the human population from the same region. Clade GRY (32.7%), G (29.2%), GR (25.3%), O (7.2%), GH (3.4%), GV (1.4%), S (0.5%), and L (0.4%) were found in environmental samples. Various lineages were identified in environmental samples; nevertheless, the highest percentages (49.4%) of the alpha variant (B.1.1.7) were detected in Austria, Liechtenstein, Slovenia, Czech Republic, Switzerland, Germany, and Italy. Other prevalent lineages were B.1 (18.2%), B.1.1 (9.2%), and B.1.160 (3.9%). Furthermore, a significant number of amino acid substitutions were found in environmental strains where the D614G was found in 83.8% of the sequences. However, the key mutations—N501Y (44.6%), S982A (44.4%), A570D (43.3%), T716I (40.4%), and P681H (40.1%) were also recorded in spike protein. The identification of the environmental belvedere of SARS-CoV-2 and its genetic signature is crucial to detect outbreaks, forecast pandemic harshness, and prepare with the appropriate tools to control any impending pandemic. We recommend genomic environmental surveillance to trace the emerging variants and diversity of SARS-CoV-2 viruses circulating in the community. Additionally, proper disposal and treatment of wastewater, sewage, and medical wastes are important to prevent environmental contamination.

## 1. Introduction

The emergence of severe acute respiratory syndrome coronavirus 2 (SARS-CoV-2) put human civilization at risk for its devastating spread. The world is facing an enormous economic loss through death, treatment cost, lockdown, travel restrictions, trade embargoes, and many other factors, but the threat might be aggravated if there is a chance of environmental contamination by the virus [1,2]. SARS-CoV-2 is mainly a respiratory virus, but it may persist and replicate in the gastrointestinal tract and shed through feces during and after the active infectious stage in humans [3,4,5,6]. Infected persons are shedding viruses through feces [6,7] and this fact was established by the presence of viral RNA in sewage, reported in the Global Initiative on Sharing All Influenza Data (GISAID) (https://www.epicov.org/, accessed on 27 July 2021) since 9 February 2020. During the SARS outbreak in 2003, about 16–73% of SARS patients exhibited diarrhea [8], and transmission occurred via water droplets from feces to the air and the environment [9]. For instance, the World Health Organization (WHO) reported an occurrence of 342 cases with 42 deaths in Hong Kong due to SARS where the virus was transmitted through a water plumbing system. However, the airborne spread of “virus-laden droplets” occurred through bathroom ventilation into the room [10]. Similarly, the SARS-CoV-2 droplet might be released through the wastewater sanitation arrangement of the building’s different floors which might cause cross-contamination [11].

The sluffing of SARS-CoV-2 in feces, urogenital, and oral washing increases the credibility of using the wastewater-based epidemiology (WBE) approach for SARS-CoV-2 surveillance and monitoring. A reverse transcriptase real-time polymerase chain reaction (rt-PCR) is used to detect N1, N2, and N3 genes of the nucleocapsid protein and the E gene of the envelope protein of the virus from wastewater [12]. As an early warning tool, the environmental surveillance of SARS-CoV-2 is crucial to detect the virus’s circulation in the human population [13,14]. WBE can be used to monitor around 2.1 billion people globally from 105,600 sewage treatment plants [15]. Earlier, environmental surveillance was found to be useful for Hepatitis A virus [16], poliovirus [14], Aichi virus [17], and norovirus [18]. Even before the identification of COVID-19 positive patients in the community, the sewage data flag out the presence of the virus. Therefore, WBE suffices to determine the circulation of coronavirus in the community [19] and the sewage network samples might be sufficient for the identification of SARS-CoV-2 RNA which act as a “mirror of the population”.

Earlier studies used WBE for the identification of SARS-CoV-2 RNA from the effluents worldwide, such as in Australia, Italy [20,21], the Netherlands [13], and Massachusetts, USA [22]. In addition, a study from Paris, France reported that SARS-CoV-2 RNA detection correlates with the number of symptomatic or asymptomatic carriers [23]. Fecal shedding of the virus dominates on oropharyngeal shedding, which is evident in China, where SARS-CoV-2 was identified in a fecal sample of a patient, rather than oropharyngeal swabs, for an unusually long period of time [24]. Another reason might be paucisymptomatic patients’ unconsciousness about their hand cleaning and mask-wearing, which subsequently promotes virus circulation to their family and ultimately affects wastewater.

Overall, the COVID-19 pandemic has a detrimental effect on public health caused by environmental risk factors [25,26,27]. So, the safe management of domestic and household waste could be critical during the ongoing pandemic. In the current situation, medical waste, both from households and hospitals, such as contaminated masks, gloves, used or expired medications, and other items, can easily be mixed with miscellaneous domestic and hospital waste [28]. Additionally, there is a data gap to understand the other possible environmental routes of transmission, such as air fomites and surface-level contamination, which steadily increases the infection rates. Since SARS-CoV-2 has been considered highly infectious among other viruses within the coronavirus family, it is important to unveil the pattern and possible pathways of environmental transmission.

Moreover, the genomic configuration of SARS-CoV-2 is attributable to inter-species transmission and adaptation into an unusual host and hence is imperious to explicate the evolutionary dynamics of the viral genome and its proclivity for differential host selection [29]. Therefore, an in-depth study of SARS-CoV-2 sequences using a robust number of sequences analyzed for phylogeny, structural, and mutational constituents might be effective to obtain a more holistic view of the genomic topographies of this virus.

Here, we conducted the study for molecular characterization of SARS-CoV-2 from environmental samples to track their relatedness and diversity with strains from community people from different geographical regions. We also attempted to illustrate the ways SARS-CoV-2 spreads from a diseased person to the sewer and vice versa.

## 2. Methodology

### 2.1. Epidemiology and Phylogeny of Environmental Strains of SARS-CoV-2

We searched the GISAID (https://www.gisaid.org/, accessed on 22 May 2021) repository for all sequences of the SARS-CoV-2 virus from different environmental sites around the world (Supplementary Table S1). We retrieved the sequences and collated them in Microsoft Excel. It should be noted that many other countries reported detection of SARS-CoV-2 without sequencing the virus. We did not include those reports in our study. We modified the time frame from 20 December 2019 to 22 May 2021. A total of 807 environmental samples have been sequenced and deposited in GISAID. We calculated the percentage of different environmental samples by country using STATA 13.0 and presented them graphically. We showed the distribution of SARS-CoV-2-positive environmental samples graphically using ArcGIS. Then, we used sequences > 29,000 nucleotides long for phylogenetic analysis using MEGA 7.0 software (MEGA, Auckland, New Zealand) [30]. We used the full-length nucleotide sequence of the Wuhan SARS-CoV-2 virus as the reference sequence (accession number NC_045512.2) [31]. We also calculated the percentages of lineages and clades of SARS-CoV-2 strains from environmental samples.

### 2.2. Mutational Analysis of SARS-CoV-2 Retrieved from the Environment

For mutation analysis, we selected 807 environmental SARS-CoV-2 sequences with more than 29,000 base pairs as described by Kiyotani, et al. [32]. We used NC_045512.2 as a reference strain across the entire set of studied genomes. We aligned the nucleotide sequences concerning the reference sequence of SARS-CoV-2 using MEGA 7.0 software and virus pathogen repositories (https://www.viprbrc.org/, accessed on 22 May 2021) [30]. We translated the individual protein of the virus into amino acid and compared it with the reference sequence using the process described by Toyoshima, et al. [33]) to identify the mutation sites of the virus. We used GISAID for the examination of the individual virus protein mutation. We graphically represented all the mutation sites in percentages.

## 3. Results

### 3.1. Frequency and Spatial Distribution of SARS-CoV-2 Sequences of Environmental Strains

**Figure 1:**
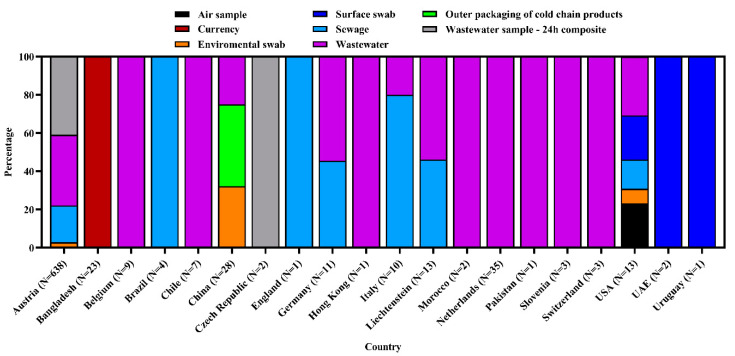
SARS-CoV-2 RNA-detected from diverse environmental samples in different countries in the world.

**Figure 2:**
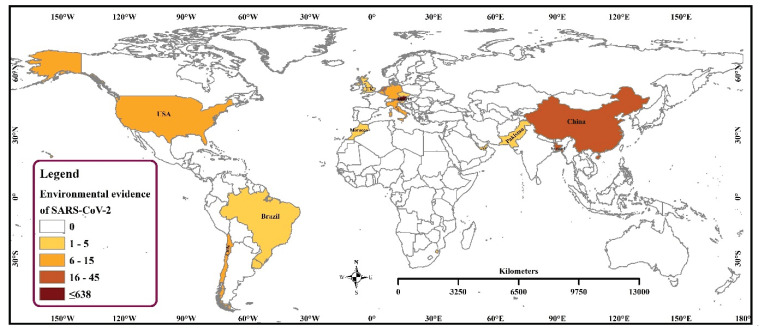
Spatial Distribution of SARS-CoV-2 RNA detected in environmental samples in the world.

We found that 20 countries reported 807 SARS-CoV-2 sequences from different environmental samples across the globe. The SARS-CoV-2 RNA has been detected in diverse samples, namely, air, currency, environmental swab, surface swab, wastewater, sewage, and the outer packaging of cold chain products. Wastewater samples were highest (40.0%) to successfully yield the virus genome sequence followed by a 24 h composite wastewater sample (32.6%), and sewage (18.5%). The highest number of samples (*n* = 638) were sequenced in Austria, followed by the Netherlands, China, and Bangladesh (Figure 1 and Figure 2).

### 3.2. Phylogenetic Analysis of SARS-CoV-2 from the Environment

**Figure 3:**
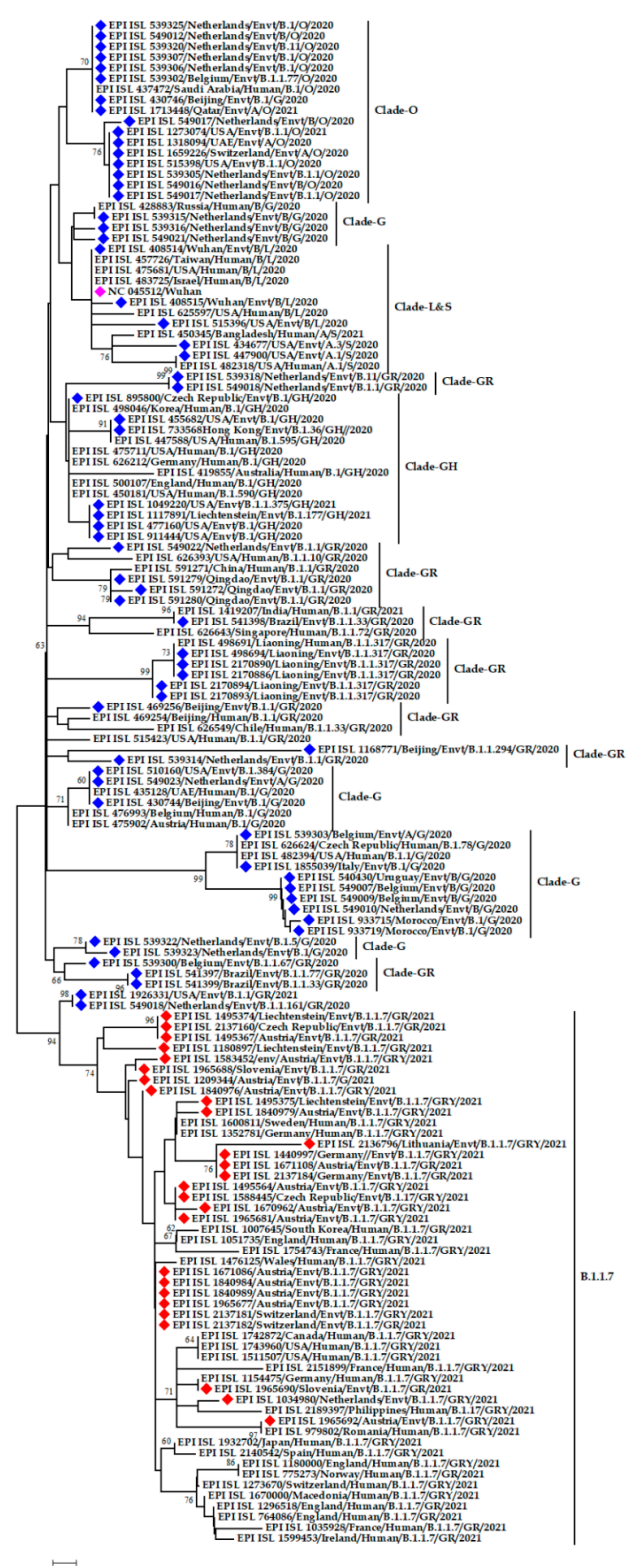
Phylogenetic analysis of SARS-CoV-2 genome sequences detected in environmental samples. Blue and red color denotes environmental strains, red color denotes the environmental alpha variants, fuchsia pink color refers to the reference sequence from Wuhan, China.

The SARS-CoV-2 strains detected from environmental samples are close congeners with the strains mostly circulating in the human population. SARS-CoV-2 strains from the Netherlands are varying in nature, forming three separate clusters. One group has a genetic resemblance with strains from Qatar and China, another with strains from the USA and Switzerland, and the rest with a human strain from Russia. On the other hand, environmental strains from the USA and Liechtenstein have the same ancestral origin. Interestingly, the human strain from India and the environmental strain from Brazil are grouped in the phylogeny. Another distinguished cluster was formed by strains from Belgium, Italy, Uruguay, and Morocco (Figure 3).

The alpha (α) variant denoted by lineage B.1.1.7 was detected in environmental samples from Austria, Switzerland, Liechtenstein, the Netherlands, Slovenia, Germany, Italy, Lithuania, and the Czech Republic. The phylogenetic tree constructed solely of the α variants from environmental strains showed that they all belong to the same lineage, though there are variations among them. Even the environmental strains from Austria have variations within themselves as they formed several small clusters rather than forming a single cluster.

The emerging α variant from environmental samples from Austria was genetically similar to human samples from Belgium, Germany, Switzerland, and Wales. Moreover, α variants from environmental strains from Austria and Germany have relations to sequences from human strains from Austria and France. Another exclusive cluster was formed by human strains from South Korea and Sweden and environmental strains from Austria and Switzerland.

**Figure 4:**
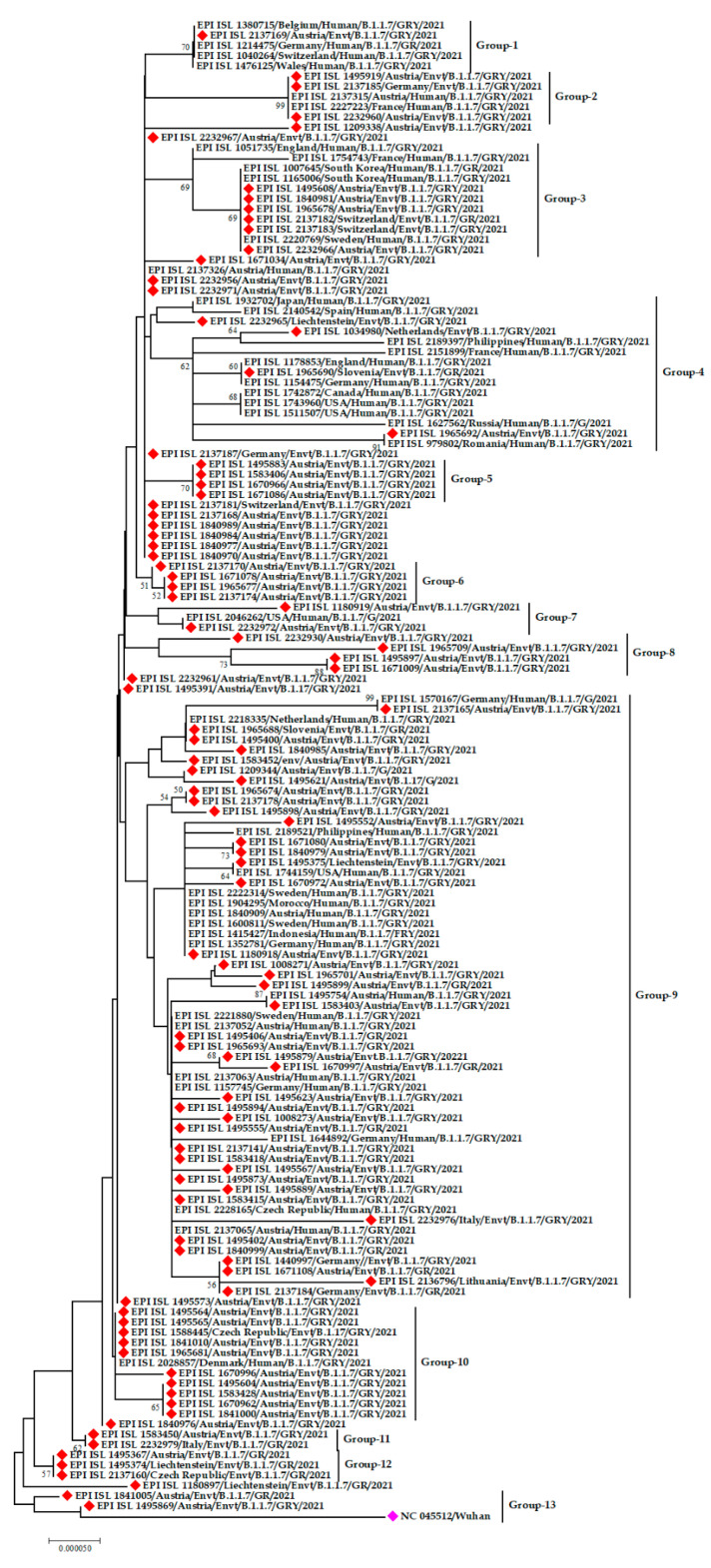
Phylogenetic analysis of α variant (B.1.1.7 lineage) of SARS-CoV-2 environmental strains. Red color denotes the environmental alpha variants, fuchsia pink color refers to the reference sequence from Wuhan, China.

Interestingly, human strains from one region were found to have genetic relatedness with environmental strains from another region. For example, the α variant of human SARS-CoV-2 from the Philippines and environmental SARS-CoV-2 from the Netherlands, human SARS-CoV-2 from England and Germany with environmental SARS-CoV-2 from Slovenia, human SARS-CoV-2 from Romania and Russia with environmental SARS-CoV-2 from Austria were found to share the same ancestral origin (Figure 4).

### 3.3. Clade and Lineage Diversity of SARS-CoV-2 Circulating in the Environment

**Figure 5:**
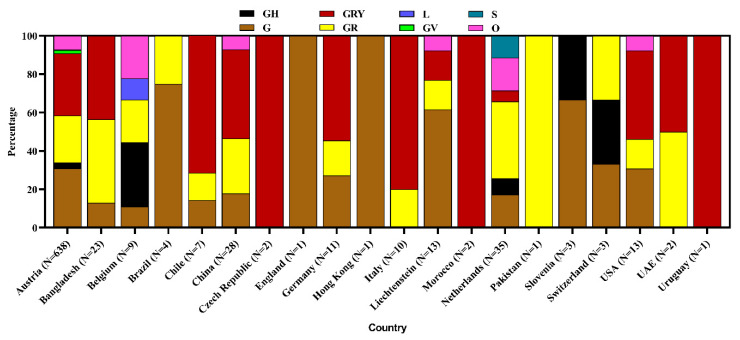
Clade diversity of SARS-CoV-2 isolated from environmental sampling.

Among the studied 807 genome sequences from different environmental samples, scientists found clade GRY (32.7%), G (29.2%), GR (25.3%), O (7.2%), GH (3.4%), GV (1.4%), S (0.5%), and L (0.4%). Austria reported the most diverse clades, among which clade GRY was highest (32.3%), followed by G (30.9%), GR (24.6%), O (7.2%), GH (2.9%), GV (1.7%), and L (0.3%) in environmental samples. The Netherlands detected six clades whereas China and Liechtenstein each detected four clades. The clade S has only been reported from the Netherlands. The overall identified clade was nonspecifically denoted as Others (O) clade (Figure 5).

**Figure 6:**
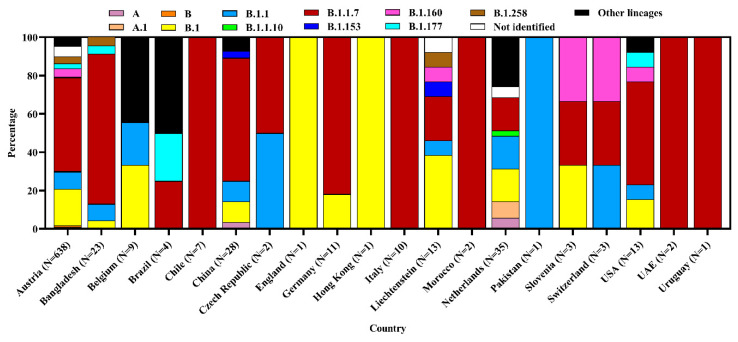
Lineage diversity of SARS-CoV-2 strains from environmental samples.

Various lineages were identified in environmental samples, but the highest percentage (49.4%) was under lineage B.1.1.7. Other prevalent lineages were B.1 (18.2%), B.1.1 (9.2%), and B.1.160 (3.9%). Furthermore, environmental strains belonged to other non-specific lineages. The most diverse (13) lineages have been reported from Austria where emerging α variant B.1.1.7 was highest at 48.9%. The USA and the Netherlands also reported diverse lineages where B.1.1.7 was 53.9% and 17.1%, respectively in each country (Figure 6).

### 3.4. Mutational Diversity in SARS-CoV-2 from Environmental Samples

**Figure 7:**
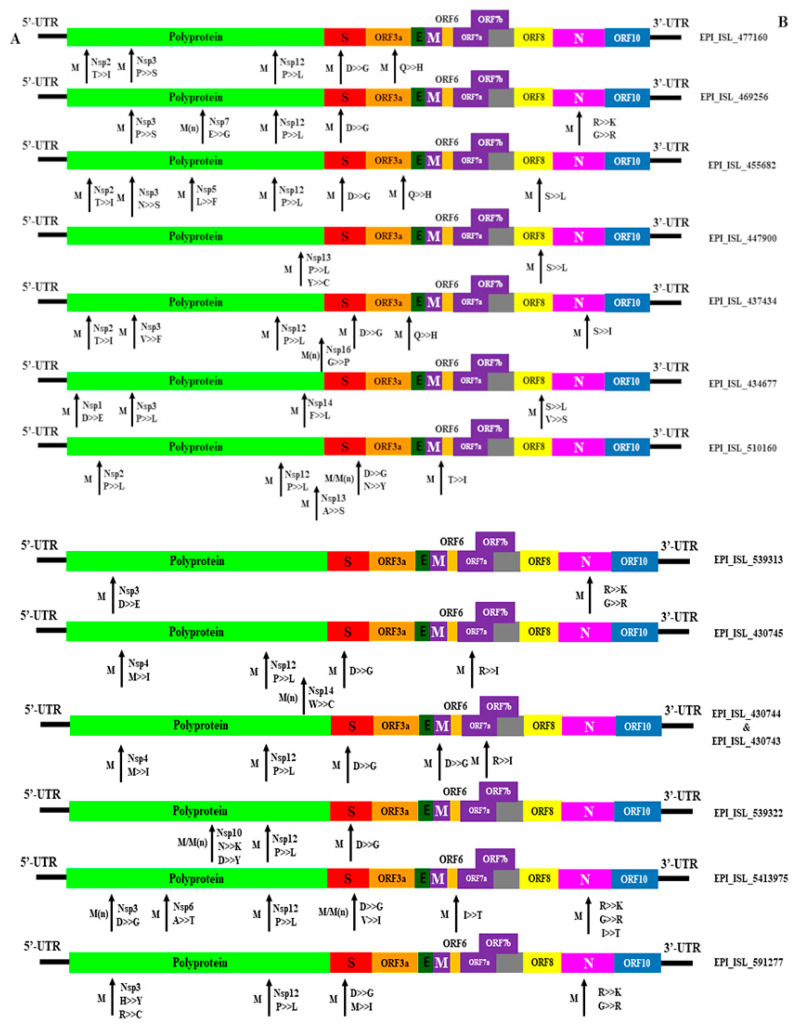
Genomic mutation analysis of environmental-originated SARS-CoV-2. >> and marking indicating the mutational changes among 12 SARS-CoV-2 (EPI_ISL_408514 is not included due to Nil mutation) strain studied in this study in comparison with the reference strain NC_045512.2, hCoV19/Wuhan/WIV04/2019. Here, M—mutation; M(n)—Novel mutation; A—genomic structure; B—accession number; protein: G—glycine, L—Leucine, I—isoleucine, P—Proline, Y—Tyrosine, W—Tryptophan, S—Serine, T—Threonine, C—Cysteine, M—Methionine, N—Asparagine, Q—Glutamine, D—Aspartate, K—Lysine, R—Arginine, H—Histidine as of 21 May 2021.

**Figure 8:**
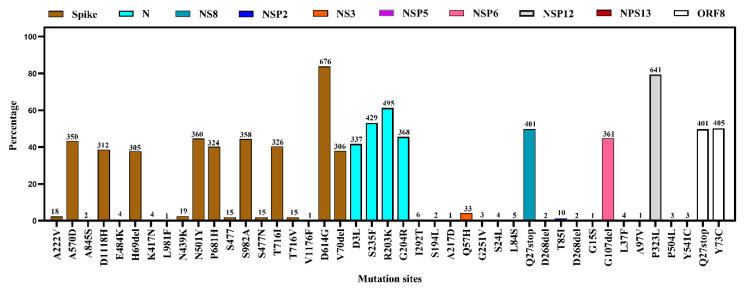
Proportion of amino acid mutations in different segments of SARS-CoV-2 environmental strains.

We observed a total of 43 mutations at different proteins of the viral genome. The most common amino acid substitutions were D614G (83.4%) followed by N501Y (44.6%), S982A (44.4%), A570D (43.3%), T716I (40.4%), and P681H (40.1%) in Spike (S) protein. N501Y and P681H are key mutations in the α variant. Another important key mutation is E484K for the South African variant which was found as 0.5% in studied genomes of the S protein. In the N protein, the highest mutation was R203K (61.3%). On the other hand, the NS3 and NS8 protein of environmental strains showed frequent mutations at Q57H and Q27stop, respectively. No mutation was recorded in NSP3 but other segments- NSP5, NSP6, NSP12, and NSP13 showed regular mutations at different points. In the ORF8 protein, mutations were found at Q27stop (49.7%) and Y73C (50.2%) (Figure 7 and Figure 8, and Supplementary File S1).

## 4. Discussion

Wastewater-based epidemiology and/or environmental surveillance for pathogens have been used previously for poliovirus [14] and currently for antimicrobial resistance (https://www.who.int/glass/en/, accessed on 25 May 2021) to help policy planning [34]. It could be useful tool for COVID-19 surveillance [35] as SARS-CoV-2 can be shed from the upper gastrointestinal and respiratory tract through feces to wastewater. The infected person can shed SARS-CoV-2 RNA in feces persistently [36]. Sewage and wastewater samples are easy to collect without any invasive sampling methods [37]. So, WBE can be a cheap alternative to screening large populations for COVID-19. WBE can detect one symptomatic/asymptomatic infected case per 100 to 2,000,000 non-infected people [15]. This approach can effectively reduce the pressure on continuous human surveillance in resource-limited settings [38]. Another important tool to monitor SARS-CoV-2 is the analysis of airborne particles [35]. These two combinedly can act as an early warning system for potential outbreaks and help to take specific preventive measures [34].

The present study focused on the environmental genomic diversity of SARS-CoV-2 reported in various countries. We found that various environmental samples such as air, currency, environmental swabs, surface swabs, sewage and wastewater, packaging of cold products, and wastewater composite samples have been contaminated with SARS-CoV-2. The number of environmental sequences of SARS-CoV-2 was reported higher in Austria than in any other country (www.gisaid.org, accessed on 22 May 2021). The number of sequencings reported varies across countries, which may be due to less interest in sequencing from environmental samples. We observed 14 countries reported the sequencing of the virus from wastewater whereas seven countries sequenced the virus from sewage samples. However, the untreated wastewater or sludge contains SARS-CoV-2 RNA in different regions, such as Italy, Spain, Australia, the Netherlands, the USA, France, and Pakistan [22,23,25,39,40]. India reported detection of SARS-CoV-2 RNA for the first time in a wastewater treatment plant in May 2020 and the genetic material increased in the samples along with increasing the number of patients in that city [41]. Similarly, Australia reported 21 positive samples out of sixty-three 24 h composite wastewater samples. They detected viral RNA in wastewater 3 weeks before the first clinical case [34]. The RNA concentration in wastewater can indicate the detection of COVID-19-confirmed cases which will be found after 4 to 7 days [25,39,40].

It is still not confirmed if the virus can be transmitted via contaminated drinking water or not. If that is the case, developing countries where wastewater treatment facilities are very scarce can face health issues from environmental contamination [41]. Moreover, contamination of the drinking and wastewater chain can expose humans to coronavirus through water [42]. Other sources of infection to humans may be in contact with the contaminated surface of inanimate objects. Another route of environmental contamination is through medical and household-generated COVID-19 wastes, such as hand sanitizer bottles, gloves, face masks, and personal protective equipment [43].

In Bangladesh, SARS-CoV-2 RNA was identified in wastewater near a COVID-19 isolation center [38] and on the banknotes [39]. A considerable number of hospitals in Dhaka, Bangladesh lacks wastewater containment and treatment facilities and directly discharge effluents to environments and ambient water bodies [38]. Moreover, occupational safety and hospital wastes are not managed properly; and healthcare workers have inadequate knowledge of hospital waste management. Hospital wastes are being disposed of in a way that is environmentally unsustainable [40]. This contributes to growing health risks to the environment as well as neighboring communities [38]. Monitoring of major drains regularly is recommended to prevent large-scale environmental contamination.

We showed the variations of the environmental strains found around the world. The environmental strains of SARS-CoV-2 were found to be genetically similar to human strains that evolved at the same period, but the country of origin was not always the same. For example, it was seen that environmental strains from the Netherlands grouped with human strains from Singapore. It indicates the movement of humans from one region to another which helps the spread of SARS-CoV-2 in the environment. This fact was evident from the similarities between the genes from the Indian wastewater treatment plant with those of Australia, China, and Turkey [41].

Austria reported the highest number of SARS-CoV-2 sequences from environmental samples. Several different cities of Austria, such as Burgenland, Carinthia, Lower and Upper Austria, Salzburg, Styria, Tyrol, Vienna, and Vorarlberg, contained the virus in their environment. They detected the virus mostly in 24 h composite wastewater samples and then in wastewater and sewage. Other European countries, such as Belgium, Czech Republic, Germany, Italy, Liechtenstein, Netherlands, Slovenia, Switzerland, and the UK also detected and sequenced the virus from sewage and wastewater. The presence of the virus in wastewater and sewage will consequently contaminate the environment and the aquatic environment. This can be a future health threat to aquatic and other mammals and sometimes to wildlife due to the possibility of adaptation of the virus into new hosts. So, it is recommended to treat wastewater and sewage properly to reduce the health threat to species other than humans.

Environmental strains of SARS-CoV-2 around the world were most of the α variant, belonging to the GRY clade and lineage B.1.1.7. The α variant increases its transmissibility and lethality over time [44]. The environmental contamination with B.1.17 is mostly reported in Austria. This variant has been circulating in the human population of Austria since March 2021 (www.gisaid.org, accessed on 22 May 2021). Several other European countries such as Slovakia, Sweden, Switzerland, and Germany had a huge number of humans infected with this variant [45,46]. So, it is not surprising at all to detect this variant from environmental samples. The only exception was found in Liechtenstein where only one human was found to be infected with this emerging variant that has been found in several environmental samples. Outside Europe, this variant of concern has also been detected in wastewater and from humans in New York, USA [47,48]. However, detection of the variant of concern, B.1.1.7, in environmental samples from various European countries implies the need for strict quarantine of human, appropriate disinfection strategies for wastewater. Without proper treatment and proper waste disposal, the virus can be retained in the wastewater [49].

The most common amino acid substitutions were D614G in the S glycoprotein of environmental strains that are commonly circulating among the clinical human samples [50,51]. This mutation increases the transmissibility and mortality rates [52]. Other important mutations, N501Y and P681H, specific for the emerging α variant of concern were found at a high percentage among the environmental strains. We found several other mutations in different protein segments in the environmental strains, but their effect on the pathogenicity and transmissibility of the virus has not been studied yet. The environmental sampling revealed those viruses have a likeness in contrast to mutation, lineage, and clade diversity with the human CoVs which ultimately indicates that wastewater sequencing might be used as a strong tool for the evidence of the virus in the community before local sequencing [53].

Earlier evidence suggests that environmental RNA degrades fast and there is no concrete indication of the persistence of the virus in marine water [54]. Contrarily, another study reported a higher concentration of the virus in the surface water and marine water where the untreated sewer drains out, with evidence of the virus in mollusks and marine sediment [55]. Further, a recent study suspected that contaminated wastewater can be a source of SARS-CoV-2 in two feral minks in their natural habitat [56]. Therefore, we could not neglect the probability of the future spillover of the virus, and we should take precautionary measures through the treatment of the raw sewer before draining into the water stream. In addition, the findings elucidated here can be logically extended to the other inanimate surfaces which might be contaminated with the virus. An inanimate surface is a common source for nosocomial infection and can act as the source of transmission of SARS-CoV-2 [56]. Hence, we need to carry out the proper personal hygiene so that the virus cannot be spilled back from the inanimate surface to humans.

## 5. Conclusions and Recommendations

SARS-CoV-2 can be spread to the environment through biomedical wastage, sewage, wastewater, inanimate surfaces, and air fomites. This has increased the risk of further spreading of the virus from the environment to other susceptible animal species. Studies detected SARS-CoV-2 RNA mostly from wastewater and sewage. The virus strains have genetic similarities with human strains. The most prevalent clades and lineages were GRY and B.1.1.7 (α-variant), respectively, in environmental samples. The strains also showed a favorable number of mutations in the spike proteins. So, screening and sequencing of SARS-CoV-2 from environmental samples is very feasible, rather than screening a large number of human samples in low-resource settings. Additionally, the screening of airborne particles could be an efficient tool and a primary warning to forecast the trend of SARS-CoV-2 infection in the community. WBE is needed to be integrated with the molecular characterization of the virus for a better understanding of the circulating strains in a community. Moreover, the extensive environmental genome sequencing of SARS CoV-2 can measure variations in viral diversity, which can designate the advent of epidemiologically or clinically related mutations. The knowledge, attitude, and practices of health care workers should be altered towards occupational safety and medical waste management. Authorities should invest in infrastructure for hospital waste and wastewater containment and treatment facilities. Wastewater and sewage water should be treated properly before discharging effluents directly to environments and ambient water bodies so that the virus will not be in its infectious form and there will be no risk of environmental contamination, and thus no virus transmission to aquatic and other mammals.

## Data Availability

Publicly available datasets were analyzed in this study are available in Supplementary Materials.

## Supplementary Materials

The following are available online at https://www.mdpi.com/article/10.3390/microorganisms9081696/s1, Excel File S1: Environmental detection of SARS-CoV-2 patient status metadata, Table S1: Acknowledgement to all researchers submitting SARS-CoV-2 sequences to the GISAID database.
