# Supplementary material for: Molecular Epidemiology of SARS-CoV-2 in Diverse Environmental Samples Globally
Source: Microorganisms. 2021 Aug 10;9(8):1696. doi: 10.3390/microorganisms9081696 (PMC8401355; doi:10.3390/microorganisms9081696)
Supplement: Supplementary file 1 [file microorganisms-09-01696-s001.zip › Supplementary File 2.pdf]

We gratefully acknowledge the following Authors from the Originating laboratories responsible for obtaining the specimens, as well as the Submitting laboratories where the genome data were generated and shared via GISAID, on which this research is based.

All Submitters of data may be contacted directly via www.gisaid.org

Authors are sorted alphabetically.

Accession ID	Originating Laboratory	Submitting Laboratory	Authors
EPI_ISL_1317742, EPI_ISL_1318094	Al Wathba Veterinary laboratory, Abu Dhabi Agriculture and Food Safety Authority (ADAFSA).	Al Wathba Veterinary laboratory, Abu Dhabi Agriculture and Food Safety Authority (ADAFSA), Abu Dhabi, United Arab Emirates. PO Box 52150.	Abdelmalik Ibrahim Khalafalla; Hassan Zackaria Ali Ishag; Mezher Mabkhout Abdulla Al Karbi and Salama Suhail Mohammed Al Muhairi* .; Saeed Mohamed Saeed Al Yammahi
EPI_ISL_1168770, EPI_ISL_1168771	Beijing Center for Disease Prevention and Control	Beijing Center for Disease Prevention and Control	Bing Lyu; Daitao Zhang; Fu Li; Lijuan Chen; Quanyi Wang; Shujuan Cui; Yang Pan; Zhaomin Feng; Zhichao Liang
EPI_ISL_437434	Bozeman Water Reclamation Facility	Wiedenheft lab, Montana State University	Anna Nemudraia; Artem Nemudryi; Blake Wiedenheft; Kevin Surya; Murat Buyukyoruk; Royce Wilkinson; Tanner Wiegand
EPI_ISL_733568	Centre for Health Protection	Hong Kong Department of Health	Alan K.L. Tsang; Dominic N.C. Tsang; Edman T.K. Lam; Peter C.W. Yip; Rickjason C.W. Chan
EPI_ISL_430743, EPI_ISL_430744, EPI_ISL_430745, EPI_ISL_430746	Chinese PLA Institute for Disease Control and Prevention	Chinese PLA Institute for Disease Control and Prevention	Lizhong Li; Peng Li , Jinhui Li
EPI_ISL_458008, EPI_ISL_458009, EPI_ISL_458010, EPI_ISL_458011, EPI_ISL_458012, EPI_ISL_458013, EPI_ISL_458014, EPI_ISL_458015			
see above	Department of Food Safety, Nutrition and Veterinary public health, Istituto Superiore di Sanita'	Department of Food Safety, Nutrition and Veterinary public health, Istituto Superiore di Sanita'	Bonadonna, L.; Bonanno Ferraro, G.; Iaconelli, M.; La Rosa, G.; Lucentini, L.; Mancini, P.; Suffredini, E.; Veneri, C.
EPI_ISL_853718, EPI_ISL_853719, EPI_ISL_853720, EPI_ISL_853722, EPI_ISL_853724, EPI_ISL_853725, EPI_ISL_853726, EPI_ISL_853727, EPI_ISL_853728, EPI_ISL_853732, EPI_ISL_853739, EPI_ISL_853740, EPI_ISL_853742, EPI_ISL_853743, EPI_ISL_853744, EPI_ISL_853745, EPI_ISL_853747, EPI_ISL_853748, EPI_ISL_853750, EPI_ISL_853755, EPI_ISL_853756, EPI_ISL_853760, EPI_ISL_853761, EPI_ISL_853763, EPI_ISL_853765, EPI_ISL_853767, EPI_ISL_853783, EPI_ISL_853785, EPI_ISL_853786, EPI_ISL_853788, EPI_ISL_853792, EPI_ISL_853796, EPI_ISL_853802, EPI_ISL_853806, EPI_ISL_853814, EPI_ISL_853929, EPI_ISL_853930, EPI_ISL_853931, EPI_ISL_853932, EPI_ISL_853933, EPI_ISL_853934, EPI_ISL_853935, EPI_ISL_853936, EPI_ISL_853937, EPI_ISL_853938, EPI_ISL_853939, EPI_ISL_853940, EPI_ISL_853941, EPI_ISL_853942, EPI_ISL_853943, EPI_ISL_853944, EPI_ISL_853945, EPI_ISL_853946, EPI_ISL_853947, EPI_ISL_853948, EPI_ISL_853949, EPI_ISL_853950, EPI_ISL_853951, EPI_ISL_853952, EPI_ISL_853953, EPI_ISL_853954, EPI_ISL_853955, EPI_ISL_853956, EPI_ISL_854220, EPI_ISL_854221, EPI_ISL_854222, EPI_ISL_854224, EPI_ISL_854225, EPI_ISL_854226, EPI_ISL_854230, EPI_ISL_854232, EPI_ISL_854234, EPI_ISL_854236, EPI_ISL_854238, EPI_ISL_854240, EPI_ISL_934633, EPI_ISL_934634, EPI_ISL_934635, EPI_ISL_934636, EPI_ISL_934637, EPI_ISL_934638, EPI_ISL_934639, EPI_ISL_934640, EPI_ISL_934641, EPI_ISL_934642, EPI_ISL_934643, EPI_ISL_934644, EPI_ISL_934645, EPI_ISL_934646, EPI_ISL_934647, EPI_ISL_934648, EPI_ISL_934649, EPI_ISL_1008280, EPI_ISL_1008281, EPI_ISL_1008282, EPI_ISL_1008283, EPI_ISL_1008284, EPI_ISL_1008285, EPI_ISL_1008286, EPI_ISL_1008287, EPI_ISL_1008288, EPI_ISL_1008289, EPI_ISL_1008290, EPI_ISL_1008291, EPI_ISL_1008315, EPI_ISL_1008316, EPI_ISL_1008317, EPI_ISL_1008318, EPI_ISL_1008319, EPI_ISL_1008320, EPI_ISL_1008321, EPI_ISL_1008322, EPI_ISL_1008323, EPI_ISL_1008324, EPI_ISL_1008325, EPI_ISL_1008326, EPI_ISL_1117775, EPI_ISL_1117778, EPI_ISL_1117782, EPI_ISL_1117783, EPI_ISL_1117784, EPI_ISL_1117785, EPI_ISL_1117787, EPI_ISL_1117790, EPI_ISL_1117795, EPI_ISL_1117802, EPI_ISL_1117805, EPI_ISL_1117807, EPI_ISL_1117809, EPI_ISL_1117888, EPI_ISL_1117891, EPI_ISL_1117893, EPI_ISL_1117895, EPI_ISL_1117896, EPI_ISL_1117911, EPI_ISL_1117913, EPI_ISL_1117917, EPI_ISL_1118083, EPI_ISL_1118084, EPI_ISL_1118085, EPI_ISL_1180886, EPI_ISL_1180887, EPI_ISL_1180888, EPI_ISL_1180889, EPI_ISL_1180890, EPI_ISL_1180891, EPI_ISL_1180892, EPI_ISL_1180893, EPI_ISL_1180894, EPI_ISL_1180895, EPI_ISL_1180896, EPI_ISL_1180897, EPI_ISL_1180898, EPI_ISL_1180899, EPI_ISL_1180900, EPI_ISL_1180901, EPI_ISL_1180902, EPI_ISL_1180903, EPI_ISL_1180904, EPI_ISL_1180905, EPI_ISL_1180906, EPI_ISL_1180907, EPI_ISL_1180908, EPI_ISL_1180909, EPI_ISL_1495362, EPI_ISL_1495363, EPI_ISL_1495364, EPI_ISL_1495365, EPI_ISL_1495366, EPI_ISL_1495367, EPI_ISL_1495368, EPI_ISL_1495369, EPI_ISL_1495370, EPI_ISL_1495371, EPI_ISL_1495372, EPI_ISL_1495373, EPI_ISL_1495374, EPI_ISL_1495375, EPI_ISL_1495376, EPI_ISL_1495377, EPI_ISL_1495378, EPI_ISL_1495379, EPI_ISL_1495380, EPI_ISL_1495381, EPI_ISL_1495382, EPI_ISL_1495383, EPI_ISL_1495602, EPI_ISL_1495603, EPI_ISL_1495604, EPI_ISL_1495605, EPI_ISL_1495606, EPI_ISL_1495607, EPI_ISL_1495608, EPI_ISL_1495609, EPI_ISL_1495610, EPI_ISL_1495611, EPI_ISL_1495612, EPI_ISL_1495613, EPI_ISL_1495614, EPI_ISL_1495615, EPI_ISL_1495616, EPI_ISL_1495617, EPI_ISL_1495618, EPI_ISL_1495619, EPI_ISL_1495620, EPI_ISL_1495621, EPI_ISL_1495622, EPI_ISL_1495623, EPI_ISL_1495624, EPI_ISL_1495625, EPI_ISL_1495894, EPI_ISL_1495895, EPI_ISL_1495896, EPI_ISL_1495897, EPI_ISL_1495898, EPI_ISL_1495899, EPI_ISL_1495900, EPI_ISL_1495901, EPI_ISL_1495902, EPI_ISL_1495903, EPI_ISL_1495904, EPI_ISL_1495905, EPI_ISL_1495906, EPI_ISL_1495907, EPI_ISL_1495908, EPI_ISL_1495909, EPI_ISL_1495910, EPI_ISL_1495911, EPI_ISL_1495912, EPI_ISL_1495913, EPI_ISL_1495914, EPI_ISL_1495915, EPI_ISL_1495916, EPI_ISL_1495917, EPI_ISL_1495918, EPI_ISL_1495919, EPI_ISL_1583435, EPI_ISL_1583436, EPI_ISL_1583437, EPI_ISL_1583438, EPI_ISL_1583439, EPI_ISL_1583440, EPI_ISL_1583441, EPI_ISL_1583442, EPI_ISL_1583443, EPI_ISL_1583444, EPI_ISL_1583445, EPI_ISL_1583446, EPI_ISL_1583447, EPI_ISL_1583448, EPI_ISL_1583449, EPI_ISL_1583450, EPI_ISL_1583451, EPI_ISL_1583452, EPI_ISL_1583453, EPI_ISL_1583454, EPI_ISL_1583455, EPI_ISL_1583456, EPI_ISL_1670962, EPI_ISL_1670963, EPI_ISL_1670965, EPI_ISL_1670967, EPI_ISL_1670968, EPI_ISL_1670996, EPI_ISL_1671077, EPI_ISL_1671078, EPI_ISL_1671079, EPI_ISL_1671080, EPI_ISL_1671081, EPI_ISL_1671091, EPI_ISL_1671092, EPI_ISL_1671095, EPI_ISL_1671104, EPI_ISL_1671105, EPI_ISL_1671110, EPI_ISL_1840969, EPI_ISL_1840970, EPI_ISL_1840971, EPI_ISL_1840972, EPI_ISL_1840973, EPI_ISL_1840974, EPI_ISL_1840975, EPI_ISL_1840976, EPI_ISL_1840977, EPI_ISL_1840978, EPI_ISL_1840979, EPI_ISL_1840980, EPI_ISL_1840981, EPI_ISL_1840982, EPI_ISL_1840983, EPI_ISL_1840984, EPI_ISL_1840985, EPI_ISL_1840986, EPI_ISL_1840987, EPI_ISL_1840988, EPI_ISL_1840989, EPI_ISL_1840990, EPI_ISL_1965673, EPI_ISL_1965674, EPI_ISL_1965675, EPI_ISL_1965676, EPI_ISL_1965677, EPI_ISL_1965678, EPI_ISL_1965679, EPI_ISL_1965680, EPI_ISL_1965681, EPI_ISL_1965682, EPI_ISL_1965683, EPI_ISL_1965684, EPI_ISL_1965685, EPI_ISL_1965686, EPI_ISL_1965687, EPI_ISL_1965688, EPI_ISL_1965689, EPI_ISL_1965690, EPI_ISL_1965691, EPI_ISL_1965692, EPI_ISL_1965693, EPI_ISL_2137164, EPI_ISL_2137165, EPI_ISL_2137166, EPI_ISL_2137167, EPI_ISL_2137168, EPI_ISL_2137169, EPI_ISL_2137170, EPI_ISL_2137171, EPI_ISL_2137172, EPI_ISL_2137173, EPI_ISL_2137174, EPI_ISL_2137175, EPI_ISL_2137176, EPI_ISL_2137177, EPI_ISL_2137178, EPI_ISL_2137179, EPI_ISL_2137180, EPI_ISL_2137181, EPI_ISL_2137182, EPI_ISL_2137183, EPI_ISL_2137184, EPI_ISL_2137185, EPI_ISL_2137186, EPI_ISL_2137187, EPI_ISL_2137188, EPI_ISL_2137189, EPI_ISL_2137190			
see above	Department of Microbiology, University Innsbruck	Bergthaler laboratory, CeMM Research Center for Molecular Medicine of the Austrian Academy of Sciences	Alexander Lercher; Alexandra Popa; Andreas Bergthaler; Anna Schedl; Bekir Erguner; Benedikt Agerer; Christoph Bock; Christoph Bock; Fabian Amman; Jakob-Wendelin Genger; Jan Laine; Lukas Endler; Maelle Le Moing; Martin Senekowitsch; Martin Senekowitsch; Michael Schuster; Michael Schuster; Petr Triska; Thomas Penz
EPI_ISL_1273074, EPI_ISL_1273075	Environmental and Global Health, University of Florida	Environmental and Global Health, University of Florida	A.T.; Bisesi; J.A. and Bisesi; J.C.; J.H.; Lednicky; Loeb; Maurelli; Rainey, A.; S.E.; Sabo-Attwood, T.
EPI_ISL_576396, EPI_ISL_583535, EPI_ISL_583536, EPI_ISL_583537, EPI_ISL_583538, EPI_ISL_583539, EPI_ISL_583540, EPI_ISL_583541, EPI_ISL_583542, EPI_ISL_583543, EPI_ISL_583544, EPI_ISL_583545, EPI_ISL_583546, EPI_ISL_583547, EPI_ISL_583548, EPI_ISL_583549, EPI_ISL_583550, EPI_ISL_583551, EPI_ISL_583552, EPI_ISL_583553, EPI_ISL_583554, EPI_ISL_583555, EPI_ISL_583556			
see above	Genome Centre	Genome Centre	A. S. M. Rubayet Ul Alam; Amina Ferdaus manami; Habiba Ibnat; Iqbal Kabir Jahid; M.Anwar Hossain; Pravas Chandra Roy; Selina Akter; Selina Akter Pravas Chandra Roy Amina Ferdaus manami Habiba Ibnat A. S. M. Rubayet Ul Alam Shireen Nigar Iqbal Kabir Jahid and M. Anwar Hossain; Shireen Nigar
EPI_ISL_804009, EPI_ISL_804010, EPI_ISL_804011, EPI_ISL_804012, EPI_ISL_804013, EPI_ISL_804014, EPI_ISL_804015			
see above	Grupo de Resistencia Antimicrobiana en bacterias patógenas y ambientales GRABPA	Facultad de Ciencias de la Vida, UNAB	Claudio Meneses; César Echeverría; Dayán Sanhueza; Eduardo Castro; Jorge Olivares; Macarena Bastías; Sebastián Wolter; Waldo Díaz
EPI_ISL_853723, EPI_ISL_853752, EPI_ISL_853762, EPI_ISL_853787, EPI_ISL_853816, EPI_ISL_854233, EPI_ISL_934650, EPI_ISL_934651, EPI_ISL_934696, EPI_ISL_1008266, EPI_ISL_1008267, EPI_ISL_1008268, EPI_ISL_1008269, EPI_ISL_1008270, EPI_ISL_1008271, EPI_ISL_1008272, EPI_ISL_1008273, EPI_ISL_1008274, EPI_ISL_1008275, EPI_ISL_1008276, EPI_ISL_1008277, EPI_ISL_1008278, EPI_ISL_1008279, EPI_ISL_1008311, EPI_ISL_1008312, EPI_ISL_1008313, EPI_ISL_1008314, EPI_ISL_1117781, EPI_ISL_1117793, EPI_ISL_1117803, EPI_ISL_1117892, EPI_ISL_1117903, EPI_ISL_1117908, EPI_ISL_1117912, EPI_ISL_1117915, EPI_ISL_1117918, EPI_ISL_1180910, EPI_ISL_1180911, EPI_ISL_1180912, EPI_ISL_1180913, EPI_ISL_1180914, EPI_ISL_1180915, EPI_ISL_1180916, EPI_ISL_1180917, EPI_ISL_1180918, EPI_ISL_1180919, EPI_ISL_1180920, EPI_ISL_1180921, EPI_ISL_1180922, EPI_ISL_1180923, EPI_ISL_1180924, EPI_ISL_1180925, EPI_ISL_1180926, EPI_ISL_1180927, EPI_ISL_1180928, EPI_ISL_1180929, EPI_ISL_1180930, EPI_ISL_1180931, EPI_ISL_1209335, EPI_ISL_1209336, EPI_ISL_1209337, EPI_ISL_1209338, EPI_ISL_1209339, EPI_ISL_1209340, EPI_ISL_1209341, EPI_ISL_1209342, EPI_ISL_1209343, EPI_ISL_1209344, EPI_ISL_1209345, EPI_ISL_1209346, EPI_ISL_1209347, EPI_ISL_1209348, EPI_ISL_1209349, EPI_ISL_1209350, EPI_ISL_1209351, EPI_ISL_1209352, EPI_ISL_1209353, EPI_ISL_1209354, EPI_ISL_1209355, EPI_ISL_1209356, EPI_ISL_1209357, EPI_ISL_1209358, EPI_ISL_1209359, EPI_ISL_1209360, EPI_ISL_1209361, EPI_ISL_1209362, EPI_ISL_1209363, EPI_ISL_1209364, EPI_ISL_1209369, EPI_ISL_1495272, EPI_ISL_1495273, EPI_ISL_1495384, EPI_ISL_1495385, EPI_ISL_1495386, EPI_ISL_1495387, EPI_ISL_1495388, EPI_ISL_1495389, EPI_ISL_1495390, EPI_ISL_1495391, EPI_ISL_1495392, EPI_ISL_1495393, EPI_ISL_1495394, EPI_ISL_1495395, EPI_ISL_1495396, EPI_ISL_1495397, EPI_ISL_1495398, EPI_ISL_1495399, EPI_ISL_1495400, EPI_ISL_1495401, EPI_ISL_1495402, EPI_ISL_1495403, EPI_ISL_1495404, EPI_ISL_1495405, EPI_ISL_1495406, EPI_ISL_1495551, EPI_ISL_1495552, EPI_ISL_1495553, EPI_ISL_1495554, EPI_ISL_1495555, EPI_ISL_1495556, EPI_ISL_1495557, EPI_ISL_1495558, EPI_ISL_1495559, EPI_ISL_1495560, EPI_ISL_1495561, EPI_ISL_1495562, EPI_ISL_1495563, EPI_ISL_1495564, EPI_ISL_1495565, EPI_ISL_1495566, EPI_ISL_1495567, EPI_ISL_1495568, EPI_ISL_1495569, EPI_ISL_1495570, EPI_ISL_1495571, EPI_ISL_1495572, EPI_ISL_1495573, EPI_ISL_1495867, EPI_ISL_1495868, EPI_ISL_1495869, EPI_ISL_1495870, EPI_ISL_1495871, EPI_ISL_1495872, EPI_ISL_1495873, EPI_ISL_1495874, EPI_ISL_1495875, EPI_ISL_1495876, EPI_ISL_1495877, EPI_ISL_1495878, EPI_ISL_1495879, EPI_ISL_1495880, EPI_ISL_1495881, EPI_ISL_1495882, EPI_ISL_1495883, EPI_ISL_1495884, EPI_ISL_1495885, EPI_ISL_1495886, EPI_ISL_1495887, EPI_ISL_1495888, EPI_ISL_1495889, EPI_ISL_1495890, EPI_ISL_1495891, EPI_ISL_1495892, EPI_ISL_1495893, EPI_ISL_1583401, EPI_ISL_1583402, EPI_ISL_1583403, EPI_ISL_1583404, EPI_ISL_1583405, EPI_ISL_1583406, EPI_ISL_1583407, EPI_ISL_1583408, EPI_ISL_1583409, EPI_ISL_1583410, EPI_ISL_1583411, EPI_ISL_1583412, EPI_ISL_1583413, EPI_ISL_1583414, EPI_ISL_1583415, EPI_ISL_1583416, EPI_ISL_1583417, EPI_ISL_1583418, EPI_ISL_1583419, EPI_ISL_1583420, EPI_ISL_1583421, EPI_ISL_1583422, EPI_ISL_1583423, EPI_ISL_1583424, EPI_ISL_1583425, EPI_ISL_1583426, EPI_ISL_1583427, EPI_ISL_1583428, EPI_ISL_1583429, EPI_ISL_1670959, EPI_ISL_1670961, EPI_ISL_1670964, EPI_ISL_1670966, EPI_ISL_1670971, EPI_ISL_1670972, EPI_ISL_1670980, EPI_ISL_1670981, EPI_ISL_1670997, EPI_ISL_1671008, EPI_ISL_1671032, EPI_ISL_1671033, EPI_ISL_1671034, EPI_ISL_1671083, EPI_ISL_1671084, EPI_ISL_1671085, EPI_ISL_1671086, EPI_ISL_1671087, EPI_ISL_1671088, EPI_ISL_1671099, EPI_ISL_1671100, EPI_ISL_1671101, EPI_ISL_1671102, EPI_ISL_1671106, EPI_ISL_1671107, EPI_ISL_1671109, EPI_ISL_1671111, EPI_ISL_1671112, EPI_ISL_1840991, EPI_ISL_1840992, EPI_ISL_1840993, EPI_ISL_1840994, EPI_ISL_1840995, EPI_ISL_1840996, EPI_ISL_1840997, EPI_ISL_1840998, EPI_ISL_1840999, EPI_ISL_1841000, EPI_ISL_1841001, EPI_ISL_1841002, EPI_ISL_1841003, EPI_ISL_1841004, EPI_ISL_1841005, EPI_ISL_1841006, EPI_ISL_1841007, EPI_ISL_1841008, EPI_ISL_1841009, EPI_ISL_1841010, EPI_ISL_1841011, EPI_ISL_1841012, EPI_ISL_1965694, EPI_ISL_1965695, EPI_ISL_1965696, EPI_ISL_1965697, EPI_ISL_1965698, EPI_ISL_1965699, EPI_ISL_1965700, EPI_ISL_1965701, EPI_ISL_1965702, EPI_ISL_1965703, EPI_ISL_1965704, EPI_ISL_1965705, EPI_ISL_1965706, EPI_ISL_1965707, EPI_ISL_1965708, EPI_ISL_1965709, EPI_ISL_1965710, EPI_ISL_1965711, EPI_ISL_1965712, EPI_ISL_1965713, EPI_ISL_2137140, EPI_ISL_2137141, EPI_ISL_2137142, EPI_ISL_2137143, EPI_ISL_2137144, EPI_ISL_2137145, EPI_ISL_2137146, EPI_ISL_2137147, EPI_ISL_2137148, EPI_ISL_2137149, EPI_ISL_2137150, EPI_ISL_2137151, EPI_ISL_2137152, EPI_ISL_2137153, EPI_ISL_2137154, EPI_ISL_2137155, EPI_ISL_2137156, EPI_ISL_2137157, EPI_ISL_2137158, EPI_ISL_2137159, EPI_ISL_2137160, EPI_ISL_2137161, EPI_ISL_2137162, EPI_ISL_2137163			
see above	Institute for Water Quality and Resource Management, Technical University Vienna	Bergthaler laboratory, CeMM Research Center for Molecular Medicine of the Austrian Academy of Sciences	Alexander Lercher; Alexandra Popa; Andreas Bergthaler; Anna Schedl; Bekir Erguner; Benedikt Agerer; Christoph Bock; Christoph Bock; Fabian Amman; Jakob-Wendelin Genger; Jan Laine; Lukas Endler; Maelle Le Moing; Martin Senekowitsch; Martin Senekowitsch; Michael Schuster; Michael Schuster; Petr Triska; Thomas Penz
EPI_ISL_854229, EPI_ISL_854237, EPI_ISL_934589, EPI_ISL_934590, EPI_ISL_934591, EPI_ISL_934592, EPI_ISL_1008202, EPI_ISL_1008203, EPI_ISL_1008204, EPI_ISL_1117897, EPI_ISL_1117898, EPI_ISL_1117899, EPI_ISL_1117902, EPI_ISL_1117905, EPI_ISL_1180831, EPI_ISL_1209268, EPI_ISL_1583430, EPI_ISL_1583431, EPI_ISL_1583432, EPI_ISL_1671089, EPI_ISL_1671094, EPI_ISL_1671096, EPI_ISL_1671097, EPI_ISL_1671103, EPI_ISL_1671108, EPI_ISL_1840897, EPI_ISL_1840898, EPI_ISL_1840899, EPI_ISL_1965668, EPI_ISL_1965669, EPI_ISL_1965670, EPI_ISL_1965671, EPI_ISL_1965672, EPI_ISL_2137191			
see above	Institute of Legal Medicine, Medical University of Innsbruck	Bergthaler laboratory, CeMM Research Center for Molecular Medicine of the Austrian Academy of Sciences	Alexander Lercher; Alexandra Popa; Andreas Bergthaler; Anna Schedl; Bekir Erguner; Benedikt Agerer; Christoph Bock; Christoph Bock; Fabian Amman; Jakob-Wendelin Genger; Jan Laine; Lukas Endler; Maelle Le Moing; Martin Senekowitsch; Martin Senekowitsch; Michael Schuster; Michael Schuster; Petr Triska; Thomas Penz
EPI_ISL_408511, EPI_ISL_408512, EPI_ISL_408513, EPI_ISL_408514, EPI_ISL_408515	Institute of Viral Disease Control and Prevention, China CDC	Institute of Viral Disease Control and Prevention, China CDC	Beiwei Ye; Dayan Wang; George F. Gao; Guizhen Wu; Juan Song; Jun Han; Mengjie Yang; Peihua Niu; Peipei Liu; Roujian Lu; Shumei Zou; Wei Zhen; Weifeng Shi; Weimin Zhou; Wenbo Xu; Wenjie Tan; Wenwen Lei; William J. Liu; Xiang Zhao; Yingze Zhao; Yuchao Wu; Zhixiao Chen; Ziqian Xu
EPI_ISL_539300, EPI_ISL_539301, EPI_ISL_539302, EPI_ISL_539303, EPI_ISL_539304, EPI_ISL_539305, EPI_ISL_539306, EPI_ISL_539307, EPI_ISL_539308, EPI_ISL_539309, EPI_ISL_539310, EPI_ISL_539311, EPI_ISL_539312, EPI_ISL_539313, EPI_ISL_539314, EPI_ISL_539315, EPI_ISL_539316, EPI_ISL_539317, EPI_ISL_539318, EPI_ISL_539319, EPI_ISL_539320, EPI_ISL_539321, EPI_ISL_539322, EPI_ISL_539323, EPI_ISL_539324, EPI_ISL_539325, EPI_ISL_549006, EPI_ISL_549007, EPI_ISL_549008, EPI_ISL_549009, EPI_ISL_549010, EPI_ISL_549011, EPI_ISL_549012, EPI_ISL_549013, EPI_ISL_549014, EPI_ISL_549015, EPI_ISL_549016, EPI_ISL_549017, EPI_ISL_549018, EPI_ISL_549019, EPI_ISL_549020, EPI_ISL_549021, EPI_ISL_549022, EPI_ISL_549023			
see above	KWR Watercycle Research Institute	Erasmus Medical Center	Bas B. Oude Munnink; Claudia M. E. Schapendonk; David Nieuwenhuijse; Frank M. Aarestrup; Gertjan Medema; Goffe Elsinga; Leo Heijnen; Lu Lu; Marion P.G. Koopmans; Matthijs Kon; Miranda de Graaf; Ray Izquierdo-Lara; Samantha Lycett
EPI_ISL_933715, EPI_ISL_933719	Laboratoire de Recherche et d'Analyses Medicales de la Gendarmerie Royale	Laboratoire de Recherche et d'Analyses Medicales de la Gendarmerie Royale	Amal Souiri; Mohammed Labioui; Momammed Labioui; Nabil Lemzaoui; S. El kabbaj; Sanaâ Lemriss; Saâd El kabbaj; elmostafa El Fahime
EPI_ISL_477160	Laboratory of Dr. John Lednicky	University of Florida	Chang-Yu Wu; John A. Lednicky; and John Glenn Morris, Jr.
EPI_ISL_541397, EPI_ISL_541398, EPI_ISL_541399	Laboratório de Virologia Comparada e Ambiental - LVCA - IOC	Laboratory of Respiratory Viruses and Measles, Oswaldo Cruz Institute, FIOCRUZ	Ana Carolina Mendonça; Camille Ferreira Mannarino; Fernando Motta; Luciana Appolinario; Marilda Siqueira; Marize Pereira Miagostovich on behalf of the Fiocruz COVID-19 Genomic Surveillance Network; Paola Resende; Tatiana Prado; Tulio Machado Fumian
EPI_ISL_541400	Laboratório de Virologia Comparada e Ambiental- LVCA- IOC	Laboratory of Respiratory Viruses and Measles, Oswaldo Cruz Institute, FIOCRUZ	Ana Carolina Mendonça; Camille Ferreira Mannarino; Fernando Motta; Luciana Appolinario; Marilda Siqueira; Marize Pereira Miagostovich on behalf of the Fiocruz COVID-19 Genomic Surveillance Network; Paola Resende; Tatiana Prado; Tulio Machado Fumian
EPI_ISL_434677	Lednicky Laboratory at	Lednicky Laboratory at Emerging	C.-Y.; Clugston; Elbadry; J.A.; J.G. Jr. and Lednicky; J.R.; M.A.; Morris; S.N.; Shankar; Wu

	Emerging Pathogens Institute	Pathogens Institute	
EPI_ISL_447900	Lednicky Laboratory at Emerging Pathogens Institute	University of Florida	Alam; C.J.; Elbadry; Gibson; J.A.; J.C.; J.G.; Lednicky; M.A. and Morris; M.M.; Stephenson
EPI_ISL_2094412, EPI_ISL_2094413, EPI_ISL_2094414, EPI_ISL_2094415, EPI_ISL_2094416	Max von Pettenkofer Institute, Virology, National Reference Center for Retroviruses, LMU Munich	Laboratory for Functional Genome Analysis; Dept. Genomics; Gene Center of the LMU Munich	Alexander Graf; Helmut Blum; Max Muenchhoff; Oliver Keppler; Stefan Krebs
EPI_ISL_1093052, EPI_ISL_1093144	Maya Juarez, Div. of Pulmonary, Critical Care and Sleep Medicine, UC Davis Medical Center	Díaz-Muñoz Lab, Department of Microbiology and Molecular Genetics, University of California, Davis	A.J. Campbell; Alexandre Tremeau-Bravard; Ana M. M. Stoian; Angela Haczku; Brandt A Robinson; Christian Sandrock; Daniel G Tompkins; David A. Coil; Greg Brennan; Ivy R. Jose; Jonathan A. Eisen; Maya Juarez; Samuel L Díaz-Muñoz; Satya Dandekar; Shefali Banerjee; Stefan Rothenburg; Stuart H. Cohen; Timothy Albertson; Tracey Goldstein
EPI_ISL_540430	Microbial Genomics Laboratory, Institut Pasteur de Montevideo, Montevideo, Uruguay	Microbial Genomics Laboratory, Institut Pasteur de Montevideo, Montevideo, Uruguay	Cecilia Salazar; Gonzalo Moratorio; Gregorio Iraola; Marianoel Pereira; Pilar Moreno
EPI_ISL_429874	Microbiology, Virology and Biemergency Laboratory-ASST FBF Sacco	Microbiology, Virology and Biemergency Laboratory-ASST FBF Sacco	Rimoldi SG; Stefani F
EPI_ISL_499042	National Institute for Biological Standards and Control (NIBSC)	National Institute for Biological Standards and Control (NIBSC)	Dimitra Klapsa; Javier Martin; Thomas Wilton
EPI_ISL_469256, EPI_ISL_591272, EPI_ISL_591273, EPI_ISL_591274, EPI_ISL_591275, EPI_ISL_591276, EPI_ISL_591277, EPI_ISL_591278, EPI_ISL_591279, EPI_ISL_591280, EPI_ISL_2170885, EPI_ISL_2170886, EPI_ISL_2170892, EPI_ISL_2170893, EPI_ISL_2170894			
see above	National Institute for Viral Disease Control and Prevention, China CDC	National Institute for Viral Disease Control and Prevention, China CDC	; 10; 4&; 7; Baoying Huang3; Cao Chen; Cao Chen3&; Chun Huang; Dayan Wang; Dayan Wang3; Dongyan Wang3; Fengqin Li6; George F. Gao; George F.Gao; George Fu Gao1; Guizhen Wu; Haibo Sun5; Hong Wang; Hong Wang3; Huilai Ma; Huilai Ma1&; Ji Wang; Ji Wang3&; Jian Cai1; Jianqun Zhang2&; Jianxing Yu1; Jingdong Song; Jun Han; Jun Meng2; Li Bai6; Li Zhao3; Liang Wang1; Lingling Mao5; Mingchun Luan2; Naiying Mao3; Ning Li6; Peihua Niu3; Qian Yang3; Ruqin Gao; Shaofeng Jiang9; Shihong Yang2; Shiwen Wang; Shuangli Zhu3; Tao Ma1; Tianjiao Ji3; Wei Yao2*; Weifeng Shi; Wenbo Xu; Wenbo Xu3*; Wenjie Tan3; Wenqing Yao5*; William J. Liu; Xiang Ren1; Xiang Zhao; Xiang Zhao3; Xiang Zhao , Lijuan Chen; Yan Zhang3; Yang Song3&; Yanhai Wang; Yanhai Wang3; Yao Meng; Yao Meng , Zhixiao Chen , Yuchao Wu; Yecheng Yao11; Ying Qin1&; Yingwei Sun5; Yong Zhang; Yong Zhang3; Yuchao Wu; Yunting Xia8; Zhaoguo Wang; Zhen Zhu3; Zhijian Bo2; Zhixiao Chen; Zhongjie Li1; Zijian Feng1*
EPI_ISL_455681	National Institute of Health, WHO Regional Reference Laboratory for Polio Eradication, Virology Department	National Institute of Health, WHO Regional Reference Laboratory for Polio Eradication, Virology Department	Alam; Angez, M.; Arshad, Y.; Badar, N.; Ikram, A.; Khurshid, A.; M. and Ahad, A.; Mahmood, N.; Salman, M.; Sharif, S.; Tamim, S.; Umair, M.
EPI_ISL_515396, EPI_ISL_515398	Nevada State Public Health Laboratory	Nevada State Public Health Laboratory	Andrew Gorzalski; Chris Laverdure; Cyprian Rossetto; David Jackson; Heather Kerwin; Joel R. Sevinsky; Natalie Crawford; Paul Hartley; Richard Tillett; Stephanie Van Hooser; Subhash C. Verma; and Mark Pandori
EPI_ISL_2250234, EPI_ISL_2250235	One Health Research Division, Center for Molecular Dynamics Nepal	One Health Research Division, Center for Molecular Dynamics Nepal	Manandhar; Napit, R.; P. and Karmacharya, D.
EPI_ISL_412425, EPI_ISL_412426	Shandong Provincial Center for Disease Control and Prevention	Beijing Institute of Microbiology and Epidemiology	Dian-Ming Kang; Li-Jun Duan; Lin-Yao; Mai-Juan Ma; Wen-Kui Sun; Xiang-Na Zhao; Xiao Wei; Xiao-Lin Jiang; Yang Hang; Zeng-Qiang Kou
EPI_ISL_1036658	Tyler Radniecki	Center for Genome Research and Biocomputing	Oregon State University TRACE Project
EPI_ISL_455682	University of Florida	University of Florida	C.-Y.; J.A.; J.G.; Lauzardo; Lednicky; M. and Morris; Wu
EPI_ISL_510160	Utah Public Health Laboratory	Pathogen Discovery, Respiratory Viruses Branch, Division of Viral Diseases, Centers for Disease Control and Prevention	Anna Uehara; Clinton R. Paden; Haibin Wang; Jing Zhang; Kerrie Franzen; Krista Queen; Mary L. Killian; Suxiang Tong; Yan Li; Ying Tao
